# Xylazine detected in unregulated opioids and drug administration equipment in Toronto, Canada: clinical and social implications

**DOI:** 10.1186/s12954-021-00546-9

**Published:** 2021-10-13

**Authors:** Jeanette M. Bowles, Karen McDonald, Nazlee Maghsoudi, Hayley Thompson, Cristiana Stefan, Daniel R. Beriault, Sarah Delaney, Ernest Wong, Dan Werb

**Affiliations:** 1 Centre on Drug Policy Evaluation, Unity Health Toronto, 209 Victoria St, Toronto, Canada; 2 Dalla Lana School of Public Health, University of Toronto, 55 College St Room 500, Toronto, ON Canada; 3 Clinical Laboratory and Diagnostic Services, Centre for Addiction and Mental Health, Toronto, ON Canada; 4 Department of Laboratory Medicine, St Michael’s Hospital, 30 Bond St., Toronto, ON Canada; 5 Institute of Health Policy, Management and Evaluation, University of Toronto, Toronto, ON Canada; 6 Division of Infectious Diseases and Global Public Health, University of California San Diego School of Medicine, 9500 Gilman Dr., La Jolla, CA USA

**Keywords:** Drug checking service, Mass spectrometry, Adulterants, Overdose, Opioids, Xylazine, Harm reduction

## Abstract

**Background:**

The North American opioid overdose crisis is driven in large part by the presence of unknown psychoactive adulterants in the dynamic, unregulated drug supply. We herein report the first detection of the psychoactive veterinary compound xylazine in Toronto, the largest urban center in Canada, by the city’s drug checking service.

**Methods:**

Toronto’s Drug Checking Service launched in October 2019. Between then and February 2021, 2263 samples were submitted for analysis. The service is offered voluntarily at harm reduction agencies that include supervised consumption services. Samples were analyzed using gas chromatography–mass spectrometry or liquid chromatography-high resolution mass spectrometry. Targeted and/or untargeted screens for psychoactive substances were undertaken.

**Results:**

In September 2020, xylazine was first detected by Toronto’s Drug Checking Service. Among samples analyzed from September 2020 to February 2021 expected to contain fentanyl in isolation (610) or in combination with methamphetamine (16), xylazine was detected in 46 samples (7.2% and 12.5% of samples, respectively). Samples were predominantly drawn from used drug equipment. Three of the samples containing xylazine (6.5%) were associated with an overdose.

**Conclusion:**

We present the first detection of xylazine in Toronto, North America’s fourth-largest metropolitan area. The increased risk of overdose associated with use of xylazine and its detection within our setting highlights the importance of drug checking services in supporting rapid responses to the emergence of potentially harmful adulterants. These data also highlight the clinical challenges presented by the dynamic nature of unregulated drug markets and the concomitant need to establish regulatory structures to reduce their contribution to overdose morbidity and mortality.

## Introduction

A variety of factors have converged resulting in the catastrophic fatal opioid overdose epidemic plaguing much of North America, which include, but are not limited to, unexpected and unknown psychoactive adulterants in the unregulated drug supply [1]. Dubbed a “crisis of historic scale,”^(p. 107)^ the opioid overdose epidemic has been largely driven by fentanyl and other high-potency opioids [2]. In 2020, opioid overdoses accounted for the loss of 69,710 lives in the United States [3] and 6214 lives in Canada [4]. Between 2019 and 2020, the City of Toronto, Canada’s largest metropolitan area, experienced at minimum a 78% increase in opioid overdose deaths [5, 6]. An opioid overdose typically results in extreme sedation, respiratory depression, and, without intervention, death. Throughout different geographic regions of North America, novel adulterants of concern have emerged that may contribute to overdose morbidity and mortality. Xylazine is one such example [7]. Xylazine is a powerful non-narcotic analgesic developed in 1962 by the Bayer Corporation, synthesized as an antihypertensive drug for general anesthesia, sedation, analgesia, and muscle relaxation in large animals. Xylazine is an agent closely aligned with clonidine in that both drugs are agonists of central alpha-2 [α-2] receptors on the brainstem. The drug is not approved for human use by any authorizing associations such as the United States’ Federal Drug Administration or Canada’s Health Canada [7]. Symptoms of ingestion include sedation, and in high doses might lead to vomiting, varying cardiovascular impacts, miosis, central and peripheral nervous system depression, transient hypertension followed by severe hypotension, and respiratory depression [7]. The drug’s effects can be potentiated when used in conjunction with powerful opioids like fentanyl and benzodiazepine-related drugs that can exacerbate respiratory depression, subsequently heightening risk of overdose and death [7, 8].

In 2000, the first documented detection of xylazine in the unregulated opioid supply occurred in the United States commonwealth of Puerto Rico [9], where it is referred to as *anestesia de caballo* [horse anesthesia] or simply *anestesia* [anesthesia]. In 2007, it was documented in Philadelphia, Pennsylvania where it is referred to as *tranq* [short for tranquilizer] [8]. Previous literature detecting xylazine has primarily been in case studies of post mortem blood samples of overdose victims or in used drug administration equipment such as syringes [8, 10, 11]. Reports from the United States in California, Colorado, Connecticut, Delaware, Illinois, Massachusetts, New York, North Carolina, and Pennsylvania [12–16], as well as in British Columbia, Canada [17], have also recently reported xylazine in the unregulated supply, suggesting the possibility of widespread adulteration and potential for further expansion of this agent in unregulated drug markets throughout North America. To that end, we report on the presence of xylazine detected by a multi-site drug checking service in Toronto, Canada, between September 2020 and February 2021. We discuss the clinical and social implications of this finding to mitigate the effects of xylazine on overdose morbidity and mortality, and other associated harms.

## Methods

The Centre on Drug Policy Evaluation in Toronto, Ontario, Canada, launched a pilot drug checking program, Toronto’s Drug Checking Service [18], in October 2019. This pilot provides people who use drugs the opportunity to submit samples anonymously and free of charge via five harm reduction agencies that have supervised consumption services. Samples can be submitted by a service user (i.e., often the person who used or plans to use the drug, or a family member, friend, drug seller, or other interested party) or a staff member from the harm reduction site on behalf of a service user. Samples submitted are accompanied by the questions “What was this sample purchased as(?)” and “Are you aware of this sample being associated with an overdose or adverse effect(?).” If “yes” was answered to the latter question, there is an open field to denote “overdose.” An overdose is defined by self-report from a service user or by staff on behalf of the service user, particularly when an overdose occurred in the supervised consumption site requiring emergency attendance by staff. Samples are analyzed using gas chromatography-mass spectrometry [GC–MS] or liquid chromatography- high resolution mass spectrometry [LC-HR/MS] at one of two clinical laboratories located at the Centre for Addiction and Mental Health or St. Michael’s Hospital. Analytic results are assessed against self-reporting of what the sample was expected to contain. To maintain anonymity, a unique identifier code is generated and linked to the submitted sample. Results are returned to harm reduction agencies within 1–2 business days. If results are sought by the service user, they can be obtained via the unique identifier code and are accompanied by tailored harm reduction strategies. Results for samples analyzed are aggregated biweekly and posted on a dedicated website, on bulletin boards at harm reduction agencies, social media, disseminated via clinical and harm reduction listservs, and alerts are distributed by Toronto Public Health. Toronto’s Drug Checking Service is the only service in Canada currently using GC–MS and LC-HR/MS for routine drug checking analysis. Due to the sensitivity of these instruments, this service is uniquely positioned to find drugs in trace amounts, which is often the case with samples provided for analysis.

For analysis, the Q-Exactive platform for high resolution mass spectrometry coupled to liquid chromatography instrumentation, and the Agilent GC6890N-MS 5975 (Agilent Technologies) mass spectrometer coupled to gas chromatography instrumentation, are used. First, compounds are physically separated through the use of liquid- or gas- chromatography columns. Following chromatographic separation, the sample is introduced into the mass spectrometer, which allows for identification of a compound with high accuracy by measuring its mass-to-charge ratio (*m/z*) and analyzing its fragmentation pattern (mass spectrum) [19]. Notably, our approaches to drug checking have high analytical specificity that can differentiate structural and/or functional analogs which allows for the identification of various types of drugs from the same family [e.g., fentanyl family] or different pharmacological classifications [e.g., central nervous system stimulants vs. depressants]. For targeted screening, the sample’s analytical findings are compared against large commercial (GC–MS) or in-house (LC-HR/MS) reference libraries. Between the inaugural month in October 2019 and February 2021, 2,263 samples were checked by Toronto’s Drug Checking Service, of which 61.0% [1381] were substances and 39.0% [882] were used drug administration equipment (e.g., used syringes, cookers, smoking equipment).

Toronto’s Drug Checking Service is supported and guided by a community advisory board of people who use drugs with diverse drug using expertise. The protocol and rationale for the evaluation of Toronto’s Drug Checking Service has been previously described [19].

## Results

Xylazine was first identified by Toronto’s Drug Checking Service in September 2020 in samples submitted with the expectation that they contained fentanyl or fentanyl with methamphetamine. From September 2020 to February 2021, 1073 samples were submitted in total. Of these samples, 610 were expected to be fentanyl and 16 were expected to be fentanyl with methamphetamine. As shown in Table 1, xylazine presented in 46 samples during this time period: 7.2% [44] of all samples expected to be fentanyl and 12.5% [2] of all samples expected to be a combination of fentanyl and methamphetamine. The majority of these samples were used drug administration equipment [84.8%]. Xylazine or other anesthetics were not expected in any submitted samples.

**Table 1:**
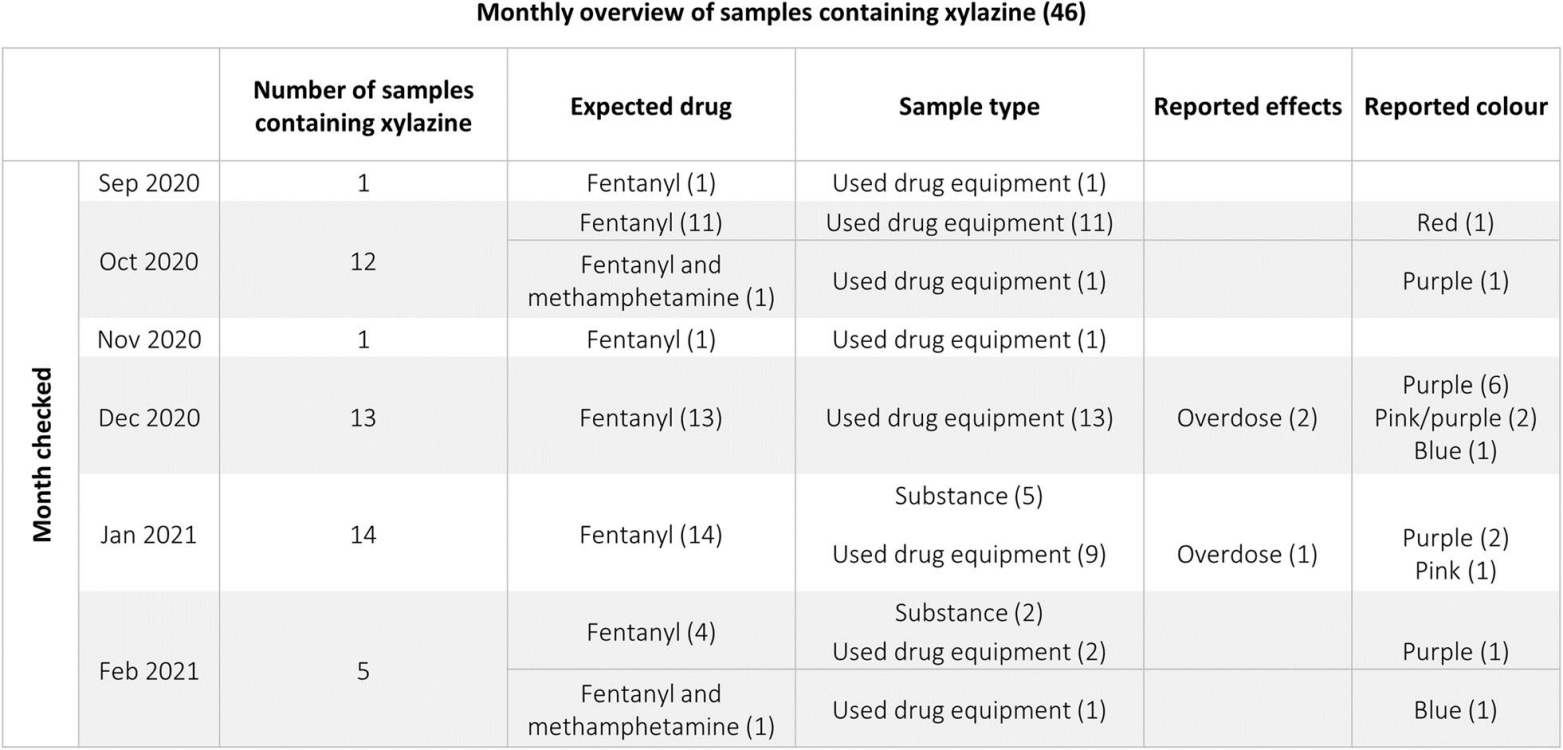
Xylazine Presence by Month, Expected Drug, Sample Type, Effects and Colour

Three of the 46 samples containing xylazine were associated with an overdose. As shown in Table 2, xylazine was most commonly found alongside ‘noteworthy drugs,’ a term we use to define substances that are associated with overdose, are highly potent or related to highly potent drugs, and/or produced undesirable effects. In xylazine-containing samples, noteworthy drugs additionally present included fentanyl [43], etizolam (benzodiazepine-related) [35], furanyl UF-17 (opioid-related) [21], and despropionyl fentanyl (4-ANPP) [20].

**Table 2:**
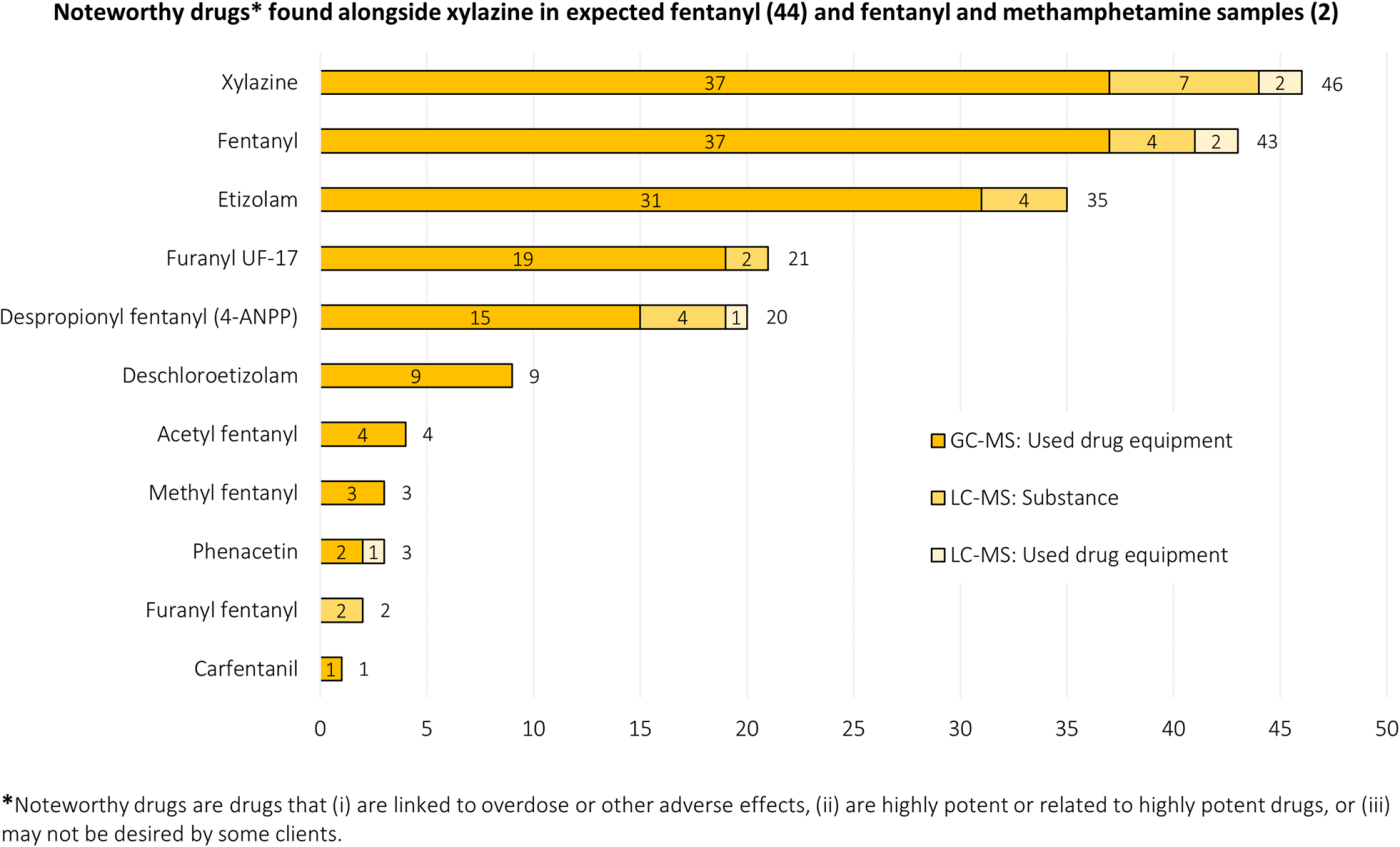
Noteworthy Drugs Alongside Xylazine

## Discussion

We report the first identification of xylazine in samples from Toronto’s unregulated drug market. Samples were all expected to contain fentanyl in isolation or in combination with methamphetamine. Xylazine has a euphoric effect that can interact with other psychoactive agents and heighten adverse health risks [1]. In humans, xylazine’s effects can last between 8 and 72 hours [7]. A primary clinical issue of concern is that a xylazine overdose will not be reversed by opioid antagonist medication naloxone. However, since it was found in samples containing opioids in our setting, the use of naloxone is warranted to reduce potential interactions. Nonetheless, in the event of an opioid and xylazine overdose, the effects of xylazine might maintain sedation, leading overdose responders to be uncertain of next steps following stabilization of biomarkers such as blood-oxygen saturation, heartbeats per minute, and unassisted respiration. Signals that xylazine overdose persists include central nervous system symptoms such as areflexia [i.e., muscles unresponsive to stimuli], hyporeflexia [i.e., muscles less responsive to stimuli], asthenia [i.e., abnormal weakness], ataxia [e.g., stumbling, slurred speech, disorientation], blurred vision, dizziness, drowsiness, dysarthria [e.g., damaged, paralyzed, or weakened speech], dysmetria [e.g., lack of coordination, damage to proprioception], faintness, somnolence [e.g., drowsiness, sedation, sleepiness], difficulty walking, and coma; respiratory depression such as apnea or shallow breathing; cardiovascular effects such as hypertension, bradycardia, tachycardia, and premature ventricular contractions; endocrine symptoms such as hyperglycemia; and miosis [i.e., constriction of the pupil or “pinned” pupils] [7]. Supportive care is the best option for treating xylazine overdose and in severe cases includes endotracheal intubation, ventilation, intravenous fluid administration, gastric lavage, activated charcoal, bladder catheterization, as well as electrocardiographic and hyperglycemia monitoring. Existing research states that hemodialysis would be ineffective at clearing xylazine due to large volume administration and distribution [10].

Another clinical issue of concern associated with xylazine injection are resulting severely infected, painful ulcers which can, in some cases, lead to amputation and even death [7, 9]. If left untreated, the extreme pain has reportedly led to the injection of xylazine directly into the ulcers, which can exacerbate the infection and ulceration [9]. A recent case study from Delaware, United States, showcased severe non-healing ulcers on a patient’s lower bilateral extremities (i.e., area of the body below the hips), and the patient confirmed injecting a mixture of fentanyl and xylazine in his legs. He noted that upon missing a vein during an intended intravenous injection, an ulceration would occur at the site of injection. The lacerations exposed fascia, tendon, muscle, and bone; and culture of the wounds identified multiple bacterial infections. Of note, the patient had left the hospital against-medical-advice for ulcers six-months prior. Upon readmission, infectious disease consultation resulting in treating the infections with intravenous antibiotics (ampicillin-sulbactam), and consultation with addiction medicine resulting in treating pain and withdrawal from opioids and xylazine with a patient-controlled analgesia pump of hydromorphone, fentanyl patches, clonidine, and benzodiazepines [16]. This case highlights the importance of an interdisciplinary, harm reduction approach to care for complex drug-related harms.

To prevent adverse outcomes associated with xylazine, ensuring that people who inject drugs are equipped with sterile injection equipment, hygienic spaces to consume drugs, injection education, and other harm reduction, clinical, and social services can mitigate these harms [20]. Of note, women and other particularly marginalized individuals who use drugs might seek to offset safety concerns resulting from xylazine’s heavy sedation by additionally consuming stimulants or using drugs alone, thereby increasing risk of harm from drug interactions and fatal overdose [21–23]. Ensuring drug consumption rooms tailor to marginalized groups could reduce this concern [20, 24]. Moreover, improving low-barrier access to opioid agonist treatments [25] and safer opioid supply programs [26, 27] could lessen harms associated with consuming drugs from the unregulated market.

The addition of xylazine in the unregulated drug market might also suggest changing drug routes from geographic manufacturing sites to the streets of Toronto. Of concern, xylazine has previously become a ‘drug of choice’ after its initial introduction into unregulated drug markets in other settings. In Puerto Rico, for example, some persons who use heroin and speedballs [i.e., heroin mixed with cocaine] have come to additionally seek xylazine [9]. In the absence of regulatory structures to reduce the harms of the unregulated drug market, raising awareness of the clinical and structural implications of adulterants can aid services supporting people who use drugs in reducing the harms of adulterants like xylazine. We note that xylazine was detected after the implementation of COVID-19 restrictions in Canada (e.g., border closures) and in Ontario (e.g., stay-at-home orders), a period that has seen elevated overdose mortality in our settings [28]. Emerging evidence suggests that the presence of adulterants in unregulated drug markets in North America is increasing since COVID-19 restrictions were put in place, as these likely impacted the production and distribution of drugs locally, nationally, and internationally [29, 30].

Our study has limitations. The samples derived from used drug equipment may have been reused, which could have led to the detection of substances from previous uses outside the time period reported herein. Additionally, it is plausible that xylazine was present in the unregulated drug market before the first detection, namely prior to Toronto’s Drug Checking Service launch in October 2019 or since then in samples not submitted for analysis to the service. Also, as overdoses indicated by service users are based on self-report, it is unknown if samples associated with an overdose were in fact potentially fatal overdose events. Regardless, report of an overdose suggests grave concern with the sample. Moreover, staff who submit samples on behalf of service users are highly trained in recognizing the signs and symptoms of an overdose, and often denote overdose based on having witnessed the overdose event connected to the sample in a supervised consumption setting.

## Conclusion

We report on the first detection of xylazine in Toronto’s unregulated drug market. This adds to emerging evidence suggesting that this substance is increasingly prevalent in unregulated drug markets across diverse North American settings during a period marked by increased adulteration and related risks [18, 28]. This is concerning given the extreme sedative effects of xylazine and the risk of overdose it presents, particularly when in combination with high-potency opioids such as fentanyl. Harm reduction services and clinicians should provide xylazine-specific modalities of care, while policymakers should seek to establish regulatory structures to mitigate the ongoing harms of dynamic unregulated drug markets, which represent the key drivers of overdose morbidity and mortality throughout North America.

## Data Availability

The datasets generated and/or analyzed during the current study are available in the Centre on Drug Policy’s Drug Checking repository: www.drugchecking.cdpe.org/reports.

## Abbreviations

GC–MS: Gas chromatography-mass spectrometry
LC-HR/MS: Liquid chromatography- high resolution mass spectrometry
